# Mechanics of post-cam engagement during simulated dynamic activity

**DOI:** 10.1002/jor.22366

**Published:** 2013-04-19

**Authors:** Clare K Fitzpatrick, Chadd W Clary, Adam J Cyr, Lorin P Maletsky, Paul J Rullkoetter

**Affiliations:** 1 Center for Orthopaedic Biomechanics, University of Denver2390 S. York St., Denver, Colorado, 80208; 2 Experimental Joint Biomechanics Laboratory, University of KansasLawrence, Kansas; 3 DePuy OrthopaedicsWarsaw, Indiana

**Keywords:** knee arthroplasty, total knee replacement, posterior-stabilized, joint mechanics, finite element analysis

## Abstract

Posterior-stabilized (PS) total knee arthroplasty (TKA) components employ a tibial post and femoral cam mechanism to guide anteroposterior knee motion in lieu of the posterior cruciate ligament. Some PS TKA patients report a clicking sensation when the post and cam engage, while severe wear and fracture of the post; we hypothesize that these complications are associated with excessive impact velocity at engagement. We evaluated the effect of implant design on engagement dynamics of the post-cam mechanism and resulting polyethylene stresses during dynamic activity. In vitro simulation of a knee bend activity was performed for four cadaveric specimens implanted with PS TKA components. Post-cam engagement velocity and flexion angle at initial contact were determined. The experimental data were used to validate computational predictions of PS mechanics using the same loading conditions. A lower limb model was subsequently utilized to compare engagement mechanics of eight TKA designs, relating differences between implants to geometric design features. Flexion angle and post-cam velocity at engagement demonstrated considerable ranges among designs (23°–89°, and 0.05–0.22 mm/°, respectively). Post-cam velocity was correlated (*r* = 0.89) with tibiofemoral condylar design features. Condylar geometry, in addition to post-cam geometry, played a significant role in minimizing engagement velocity and forces and stresses in the post. This analysis guides selection and design of PS implants that facilitate smooth post-cam engagement and reduce edge loading of the post.

The posterior cruciate ligament (PCL) is a primary contributor to anterior knee stability. During total knee arthroplasty (TKA), the PCL is often sacrificed. Posterior-stabilizing (PS) TKA components, which are designed to guide anteroposterior (AP) motion and rollback, are employed in lieu of the PCL. PS designs typically include a tibial post and femoral cam that engage as the knee flexes. Many patients receiving PS TKAs report a clicking sensation when the post and cam engage, likely due to excessive post-cam impact velocity. In a retrieval study of 23 components, seven posts (30%) exhibited severe wear, while two required early revision due to wear.^1^ While uncommon (≤1% for most TKA designs), fracture of the tibial post, necessitating surgical intervention, has been reported.^1^–^6^ Understanding the mechanics of post-cam engagement will aid in design and selection of implants that improve patient comfort and reduce post wear and risk of fracture.

A number of investigations into post-cam engagement mechanics were previously reported. The majority evaluated the influence of post-cam geometry on post wear, contact mechanics, or tibiofemoral (TF) kinematics.^1^–^10^ A variety of approaches were implemented, including implant retrievals, fixtured-only mechanical testing of devices, in vitro testing of devices, in vivo evaluation, and computational modeling. Retrieval studies compared wear location and severity of the tibial insert across different designs.^1^–^11^ Several investigators used electronic sensors or pressure-sensitive film to measure contact area, pressure, or forces at the post-cam interface. These studies compared contact mechanics of different designs in fixtured-only tests^7^–^10^ or in cadaveric experiments.^12^,^13^ Others used computational models to predict the effect of post-cam design on contact mechanics.^8^,^9^ However, these mechanical, in vitro, and in silico investigations typically studied component performance in a small number of static positions, and thus did not report engagement velocity.

Several studies employed in vivo imaging techniques. Fluoroscopy, typically integrated with 3D models of the TKA components and used to track the relative poses between components during dynamic activity, has been used to calculate the timing of post-cam engagement or describe the effect of post-cam engagement on tibiofemoral kinematics.^16^,^17^ These studies generally estimate the point of post-cam contact by calculating the closest point between the cam and post (e.g., Shimizu et al.^17^ assumed that a distance ≤0.5 mm indicated engagement), while others capture images at discrete flexion angles, requiring interpolation between images (Suggs et al.^18^ reported flexion angle of engagement based on interpolation of fluoroscopic images at 15° increments). While TF kinematics can be reasonably estimated from these analyses, contact mechanics have not been predicted from fluoroscopy alone. To overcome the difficulty of identifying post-cam contact, a combination of fluoroscopy and finite element (FE) models was employed to assess TF and post-cam articulations.^19^ Fluoroscopic kinematics were used to drive an FE model and predict the contact location on the post-cam interface. However, likely due to accuracy limitations in kinematic predictions from fluoroscopy, contact area or stress was not reported.

The dynamics of post-cam engagement are critical to joint stability, insert wear and fracture risk; the engagement velocity impacts the forces, stresses and potential longevity of the post. Excessive impact velocity likely causes the clicking sensation felt by many PS TKA patients at engagement. Thus far, however, engagement velocity has not been reported for PS TKAs. Also, investigations into the influence of design on post-cam mechanics have focused mostly on the post-cam design^1^–^10^; the influence of TF condylar geometry is largely ignored. We hypothesized that TF condylar geometry also plays an important role, including sagittal plane radius of the femoral component, post location relative to the insert dwell point, and the location of the center of the femoral radius relative to the cam. We evaluated the effect of implant design on contact mechanics and engagement dynamics during dynamic activity. A computational model was first validated against in vitro experiments, and subsequently simulation of a dynamic squat was used to compare post-cam engagement mechanics of eight TKA designs.

## MATERIALS AND METHODS

**Figure 1:**
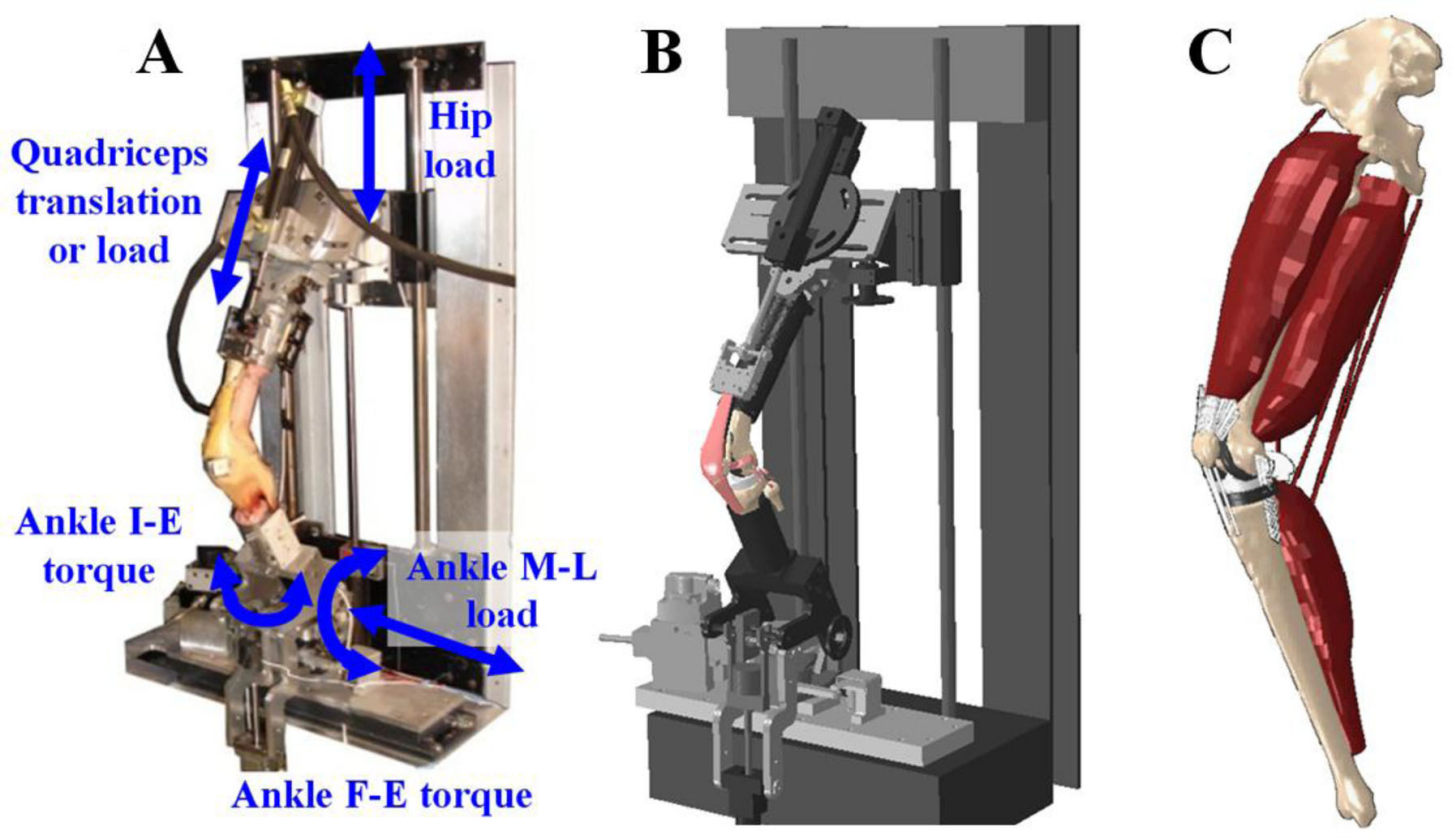
In vitro experiments of implanted TKA knees were performed in the KKS (A); computational simulations of virtually implanted TKA knees were performed in an FE model of the KKS (B) and a lower limb (C).

A TKA knee system (Sigma™, DePuy, Warsaw, IN) was tested in vitro in a knee simulator. TKA was performed on four fresh-frozen, healthy cadaveric knees by an experienced surgeon. Each implanted specimen was mounted into the Kansas knee simulator (KKS), and a deep knee bend was performed from 10° to 110° flexion. The KKS is a dynamic simulator, with loads applied at the hip, ankle, and quadriceps; the knee remains unconstrained in all six degrees-of-freedom (6-DOF)^20^ (Fig. 1). Medial-lateral (ML) translation and all rotational DOFs at the ankle were unconstrained. Light emitting diode markers were rigidly fixed to the tibial and femoral fixtures and used to track 6-DOF kinematics with a motion analysis system (Northern Digital, Inc., Waterloo, CA), with an accuracy of 0.05° for a 10° rotation and 0.03 mm for a 10 mm translation^21^. A hand-held digitizer was used to identify the position of the TKA component relative to the rigid body markers. Dimple points were machined into the components to ensure accurate component placement in subsequent computational analysis.

Combining the position data from the motion system with computer-aided-design (CAD) models from the implant manufacturer, the 3D kinematics between the femoral component and tibial insert were calculated throughout the activity. Relative 3D kinematics were used to estimate post-cam velocity and the flexion angle at the point of engagement. Post-cam velocity was calculated by identifying the points on the post and cam that came into initial contact, calculating the distance between these points throughout the cycle, and reporting the change in distance as a function of change in flexion angle. A flexion-extension cycle took ∼20 s, so velocity was measured as mm/° to normalize for flexion rate of (°/s).

A previously developed FE model of the KKS was virtually implanted with the same TKA components as the cadaveric tests. The model included femur, tibia, and patella, TKA components, TF ligaments, extensor mechanism, quadriceps muscles, and mechanical actuators (used to apply loads at the hip, ankle, and quadriceps)^22^ (Fig. 1). Bone and femoral components were meshed with triangular shell elements; polyethylene patellar and tibial components were represented by eight-noded solid hexahedral elements. Polyethylene components were modeled as fully deformable nonlinear elastic-plastic polyethylene (initial *E* = 572 MPa, *ν* = 0.45).^23^ For computational efficiency, bone and the femoral component were considered rigid. A friction coefficient of 0.04 was applied between articular surfaces.^23^ Replicating the loading condition in the in vitro tests, the ankle was fixed in AP and superior–inferior (SI) DOFs, with ML motion and all rotational DOFs unconstrained. A vertical force was applied to the hip, which was fixed in ML, AP, varus-valgus (VV) and internal-external (IE) DOFs, and unconstrained in flexion-extension (hip flexion was controlled through loading of the quadriceps). This resulted in a fully unconstrained six-DOF knee joint. Six-DOF kinematics during the simulation were recorded. Post-cam velocity and flexion angle at engagement were compared with in vitro measurements to verify the accuracy of post-cam mechanics predicted by the computational model.

To overcome some of the limitations of experimental simulators in reproducing in vivo dynamic activities, the model’s boundary conditions were adapted to better represent physiological loading at the knee.^24^ This included prescribing hip motion relative to the ankle and modifying applied loads at the hip and ankle (vertical hip force, ankle flexion-extension, and IE moments) to create a loading condition at the TF joint that reproduced experimental joint load measurements. In general, requiring the AP hip position to remain fixed directly above the ankle during a deep squat increases the quadriceps and patellofemoral forces, hence allowing AP hip motion in the model improves the fidelity of the joint forces.^24^ Loads at the hip, ankle, and quadriceps were adapted, using a previously described feedback control system integrated with the model^24^–^25^ to reproduce in vivo TF loads measured from a telemetric TKA patient performing a deep knee bend.^26^ Eight commercial TKA devices from five manufacturers (NexGen™; Zimmer, Warsaw, IN; Vanguard™; Biomet, Warsaw, IN; Sigma™, Attune™; DePuy, Warsaw, IN; Triathlon™, Scorpio™; Styker, Kalamazoo, MI; Genesis II™, Journey™; Smith & Nephew, Memphis, TN) were virtually implanted in the lower limb. Components were aligned such that a consistent joint line was maintained for all analyses with a posterior tibial slope as indicated by manufacturer guidelines. Each component was positioned such that their dwell points were aligned along the same ML axis; the insert dwell was determined by allowing the femoral component to settle into a neutral position under a compressive load at full extension. CAD geometries were obtained from the manufacturer or reverse-engineered from laser scans of the components. The deep knee bend simulation was performed for each design, allowing comparison using the same loading conditions (Fig. 1).

Post-cam velocity and engagement angle were calculated for each device throughout the knee bend simulation. Additionally, contact mechanics (area, peak pressure, and location) on the post and medial and lateral condyles, internal stress in the tibial post, forces acting on the post, and AP femoral translations with respect to the tibia were recorded for each design. Medial and lateral AP translations were calculated by identifying the lowest medial and lateral points, respectively, on the femoral articular geometry along the SI axis of the tibia.^27^ Stress in the post was reported as peak von Mises stress and as the 90th percentile von Mises stress—that is, 10% of the post (by volume) was stressed above this value.

**Figure 2:**
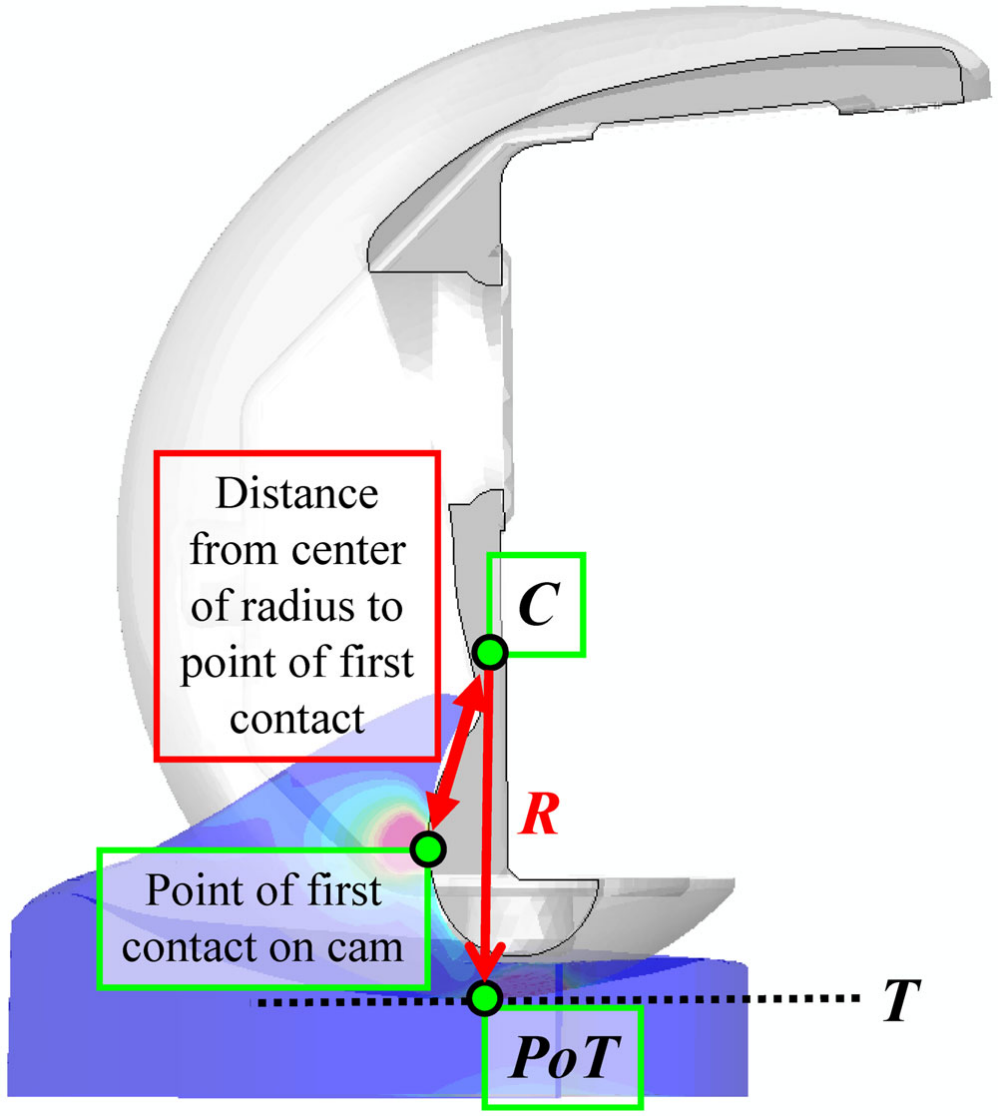
Measurements of the TKA geometries included the radius of the condyles at post-cam engagement (*R*), the center of radius at engagement (*C*), and the sagittal plane distance from *C* to the initial point of post-cam contact. *C* was found by calculating the tangent (*T*) to the femoral articular geometry lowest point; in the sagittal plane, *C* was on a perpendicular to *T*, and a distance *R* from the point of tangency (*PoT*).

Measurements were performed to quantitatively compare geometric features among designs. The femoral sagittal plane radii were measured with respect to flexion angle—of particular interest was the radius at the point of post-cam engagement (radius at engagement, *R*). The center of the radius at engagement (*C*) was found by calculating the tangent (*T*) to the femoral articular geometry at the lowest point on the femur (relative to the tibial SI axis). *C* was perpendicular to *T* and a distance *R* from the point of tangency (PoT). The distance from *C* to the point of first contact on the cam was also calculated (Fig. 2). This was measured in the sagittal plane. ML width of the post and cam, AP position of the post relative to the insert dwell point, and the SI height of initial contact relative to the insert dwell point were measured. Linear correlation coefficients, assuming a significance level of 0.05, were calculated between post-cam mechanics (engagement velocity, flexion angle at engagement) and geometric features to determine which design factors contributed to the mechanics of post-cam engagement.

## RESULTS

**Figure 3:**
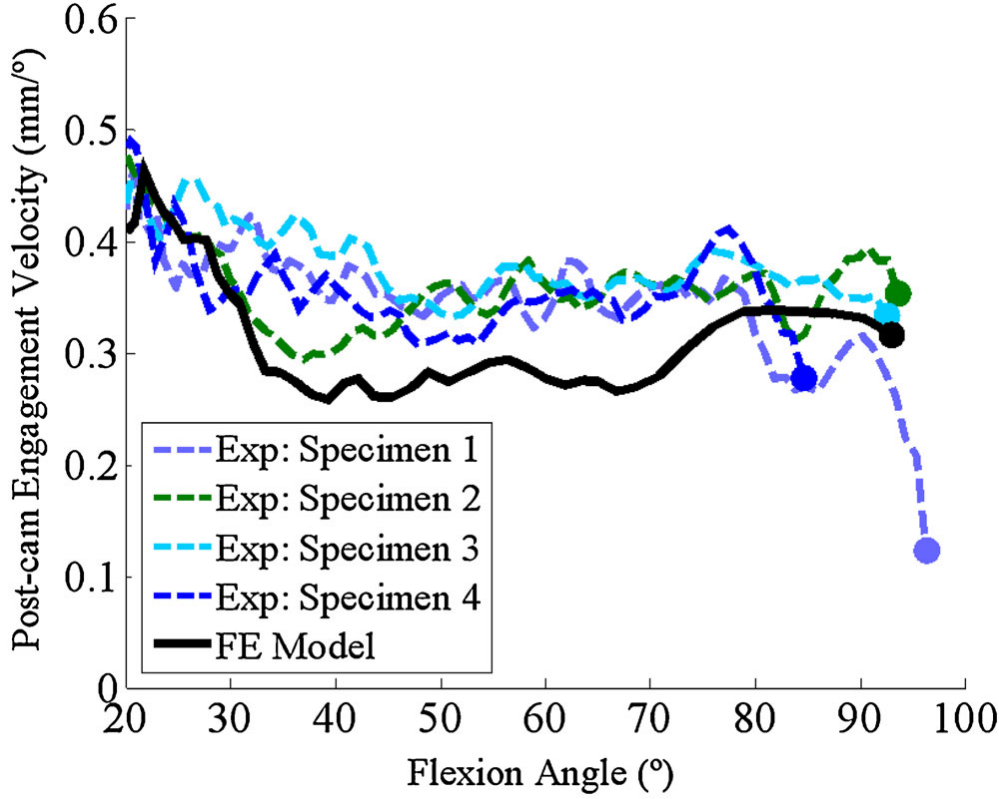
Comparison between experimental and computational post-cam engagement velocity and engagement flexion angle predictions. Markers indicated the point of initial engagement.

Flexion angle at engagement was 92° ± 5° and 93° for the experimental and computational simulations, respectively. Post-cam engagement velocity was 0.27 ± 0.1 mm/° and 0.32 mm/° for the experimental and computational simulations, respectively (Fig. 3).

**Figure 4:**
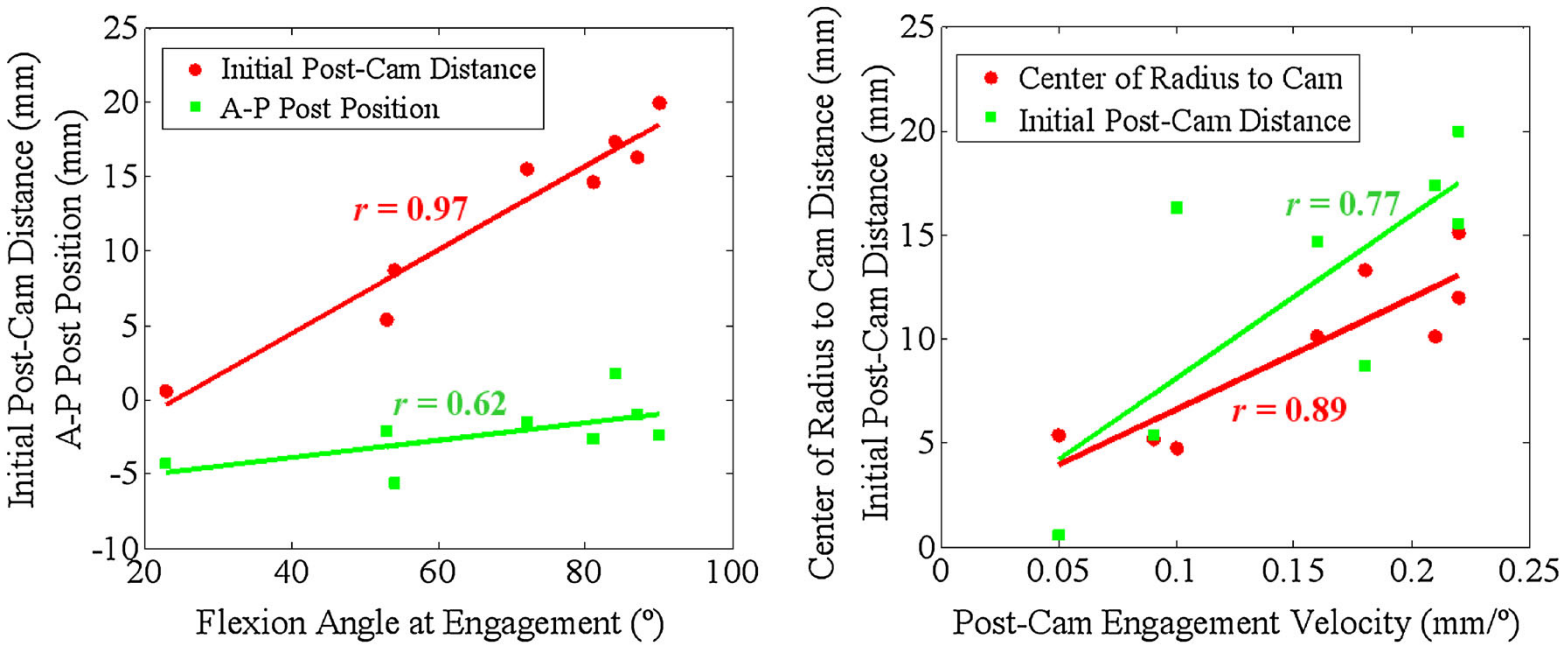
Left: Correlation between flexion angle at engagement with the initial post-cam distance, and AP position of the posterior surface of the post. Right: Correlation between post-cam engagement velocity with distance from the center of the condylar radius of curvature at engagement to the point of first contact on the cam, and the initial distance between the post and the cam.

The flexion angle at engagement ranged from 23° to 89° among the eight designs, and correlated well (*r* = 0.97) with the initial distance between the post and the cam (at the start of the squat cycle) and to a lesser non-significant extent the AP position of the posterior surface of the post (*r* = 0.62, *p* = 0.1; Fig. 4).

**Figure 5:**
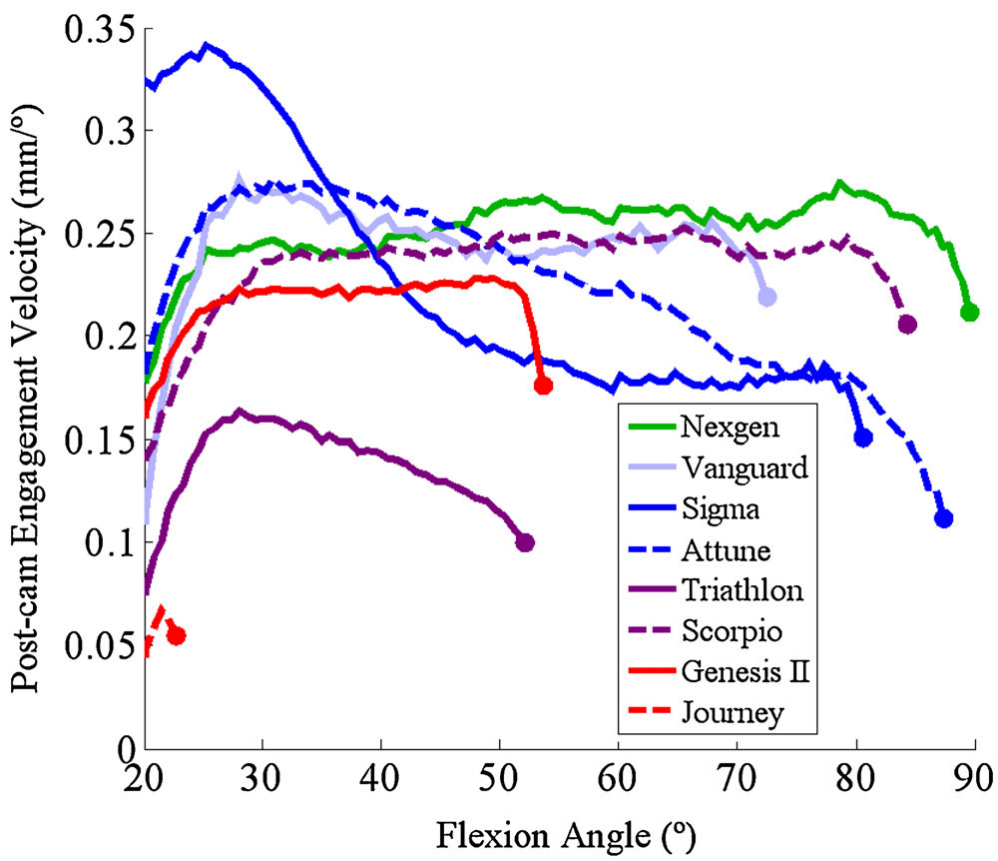
Post-cam velocity for 8 TKA designs during a squat activity (markers indicate velocity at the instant of post-cam engagement).

**Figure 6:**
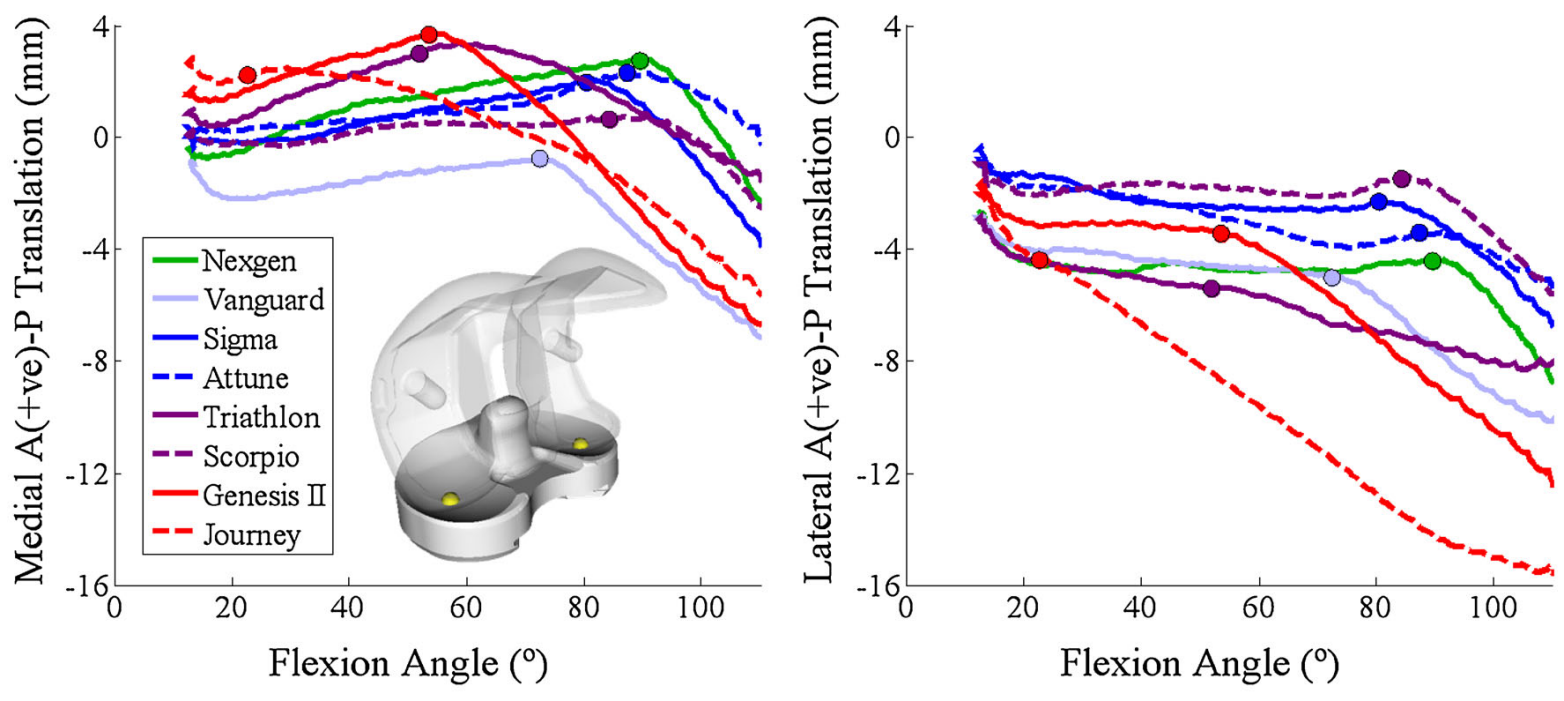
AP translation of the lowest points of the femoral medial and lateral condyles for eight TKA designs (markers indicate the point of post-cam engagement).

Post-cam velocity at engagement varied considerably among implants, ranging from 0.05 to 0.22 mm/° (Fig. 5). Post-cam velocity correlated well with condylar geometric measurements; velocity at engagement correlated with the distance from the center of the condylar radius at engagement to the point of first contact on the cam (*r* = 0.89) and also with the initial distance between the post and the cam (*r* = 0.77; Fig. 4). TKA designs with the largest distance (Vanguard, NexGen, Scorpio) had the highest impact velocity; conversely, designs with the smallest distance (Journey, Triathlon, Attune) had the slowest velocity. General trends existed between post-cam contact velocity and AP lowest point kinematics; high engagement velocity resulted in an abrupt change in lowest point kinematics at engagement, while low velocity resulted in more gradual posterior translation of the condyles during and after engagement (Fig. 6). The exception was Scorpio, which engaged at 0.21 mm/° and demonstrated gradual rollback after engagement. The slopes of medial and lateral AP translations, plotted with respect to flexion angle (Fig. 6) and the change in slope were calculated before and after engagement for each design. Correlation coefficients were calculated between the slopes (and change in slope) and engagement velocity. Velocity was correlated with the slope after post-cam engagement (*r* = −0.76; *r* = −0.70, for medial and lateral translations, respectively), in addition to the change in slope (*r* = 0.75; *r* = 0.90, for medial and lateral translations, respectively).

**Figure 7:**
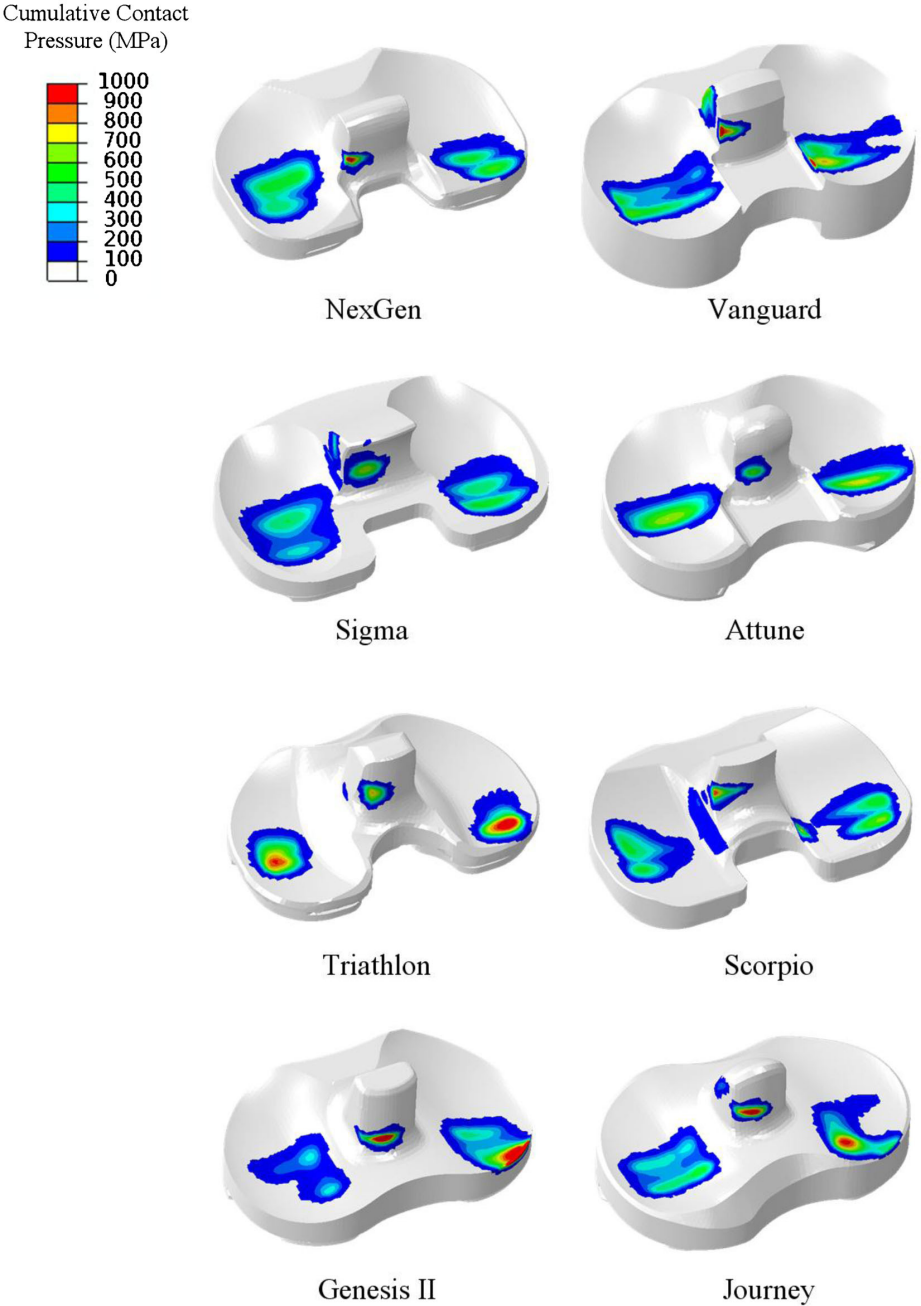
Cumulative contact pressure over the squat cycle (from 10° to 110° flexion) for eight TKA designs implanted into a right knee.

**Figure 8:**
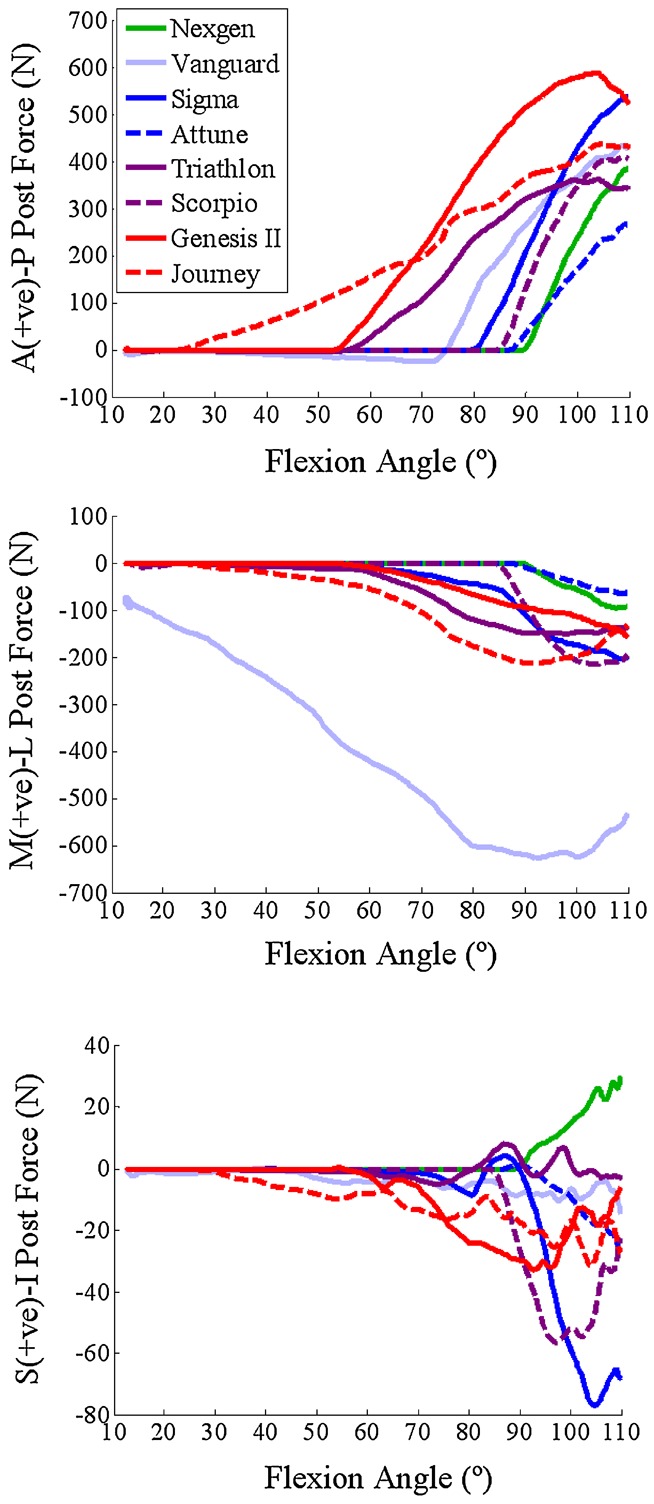
AP (top), ML (center) and SI (bottom) force on the tibial post for eight TKA designs. Force directions are described as acting on the post.

External femoral torque imposed by the loading condition resulted in loading of the medial edge of the post and high ML force in Vanguard, Journey, Scorpio, and Sigma; Figs. 7 and 8). For Vanguard, the femoral box contacted with the anterior face of the post in early flexion, resulting in a net posterior force on the post until the cam made contact in later flexion. ML force on the post correlated well (*r* = 0.78) with the ML spacing between the post and femoral box; designs with a wider gap (Attune, NexGen) had lower ML force. An association between AP position of the posterior post surface and peak AP force on the post was found, but did not reach significance (*r* = 0.57, *p* = 0.1). The SI position (relative to the insert dwell point) at which initial contact occurred was correlated with flexion angle at engagement (*r* = −0.73) and post-cam velocity at engagement (−0.72); superior contact position on the post was related to an earlier engagement and slower velocity.

**Figure 9:**
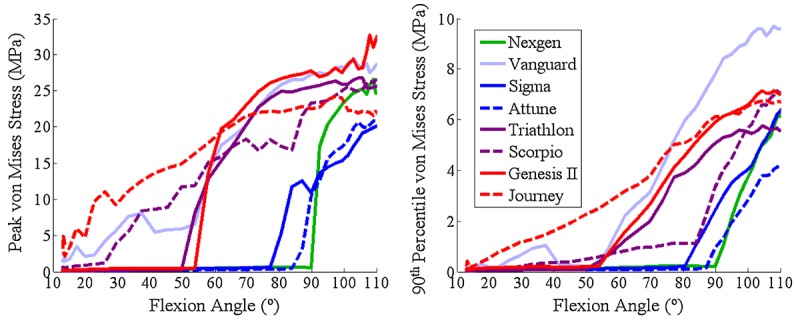
Peak von Mises stress in the tibial post (left); 90th percentile stress in the tibial post—that is, 10% of post, by volume, was stressed above this value (right).

Peak von Mises stress in the post ranged from 20 to 33 MPa, with Attune, Sigma, and Journey maintaining the lowest, and Genesis II and Vanguard creating the highest stress. The 90th percentile value ranged from 4 (Attune) to 9.5 MPa (Vanguard) in deep flexion (Fig. 9). Higher von Mises stress was mildly correlated with higher engagement velocity (*r* = 0.54), larger distance from the center of the radius at engagement to the point of first contact on the cam (*r* = 0.51), and narrower ML post-femoral gap (*r* = −0.59), although none of these relationships reached a level of significance (*p* > 0.05).

## DISCUSSION

Prior studies investigating post-cam mechanics under dynamic conditions measured sagittal plane angle of the patellar tendon^16^ or post-cam engagement flexion angle^17^–^28^ but did not report post-cam contact mechanics or engagement velocity. Those studies that measured contact area, pressure, and forces on the post were limited to a small number of static poses.^7^,^10^ Additionally, mechanical or in vitro experiments typically did not assess or compare devices under physiological loading; Nicholls et al.^13^ tested cadaveric knees during passive motion, with a 40 N load applied to the quadriceps in deepest flexion; Nakayama et al.^10^ and Akasaki et al.^7^ evaluated components under a 500 N posterior force, without any compressive force applied to the TF articular surface. We present post-cam mechanics and engagement velocity under dynamic physiological conditions.

A number of studies investigated the impact of component design on post-cam mechanics, comparing flat-on-flat and curve-on-curve geometries^9^ or describing differences in contact mechanics of commercial devices based purely on post-cam geometry.^10^ However, our study highlights the importance of tibiofemoral articulation on post-cam mechanics during dynamic activity. Femoral condyle geometry (distance from the center of radius at engagement to the point of initial contact on the cam) was strongly related to engagement velocity. Additionally, flexion angle of engagement was more strongly related to the initial post-cam distance than the position of the post alone. The relationship between engagement dynamics and design metrics illustrate the design decisions that can reduce engagement velocity. Smaller distance from the center of the condylar radius of curvature at engagement to the point of first contact on the cam and a smaller initial post-cam distance were both associated with lower engagement velocity. Lower velocity typically resulted in smoother AP kinematics, facilitating a more stable transition into deep flexion and eliminating a potential source of instability.

Our study also reiterates the influence of post design features that have been described in prior studies. Some devices are designed with an ultra-constraining post to resist VV deformity and limit tibial rotation. Nakayama et al.^10^ determined that robustness of contact mechanics to IE rotation depends on the width of the post relative to the cam in agreement with predictions from our models, which demonstrated that designs with narrower spacing between the post and the femoral box were sensitive to edge loading and high ML force. Also, peak contact pressure on the post and internal post stresses were better correlated with ML than AP force, suggesting that a design that reduces or eliminates edge loading will have improved wear resistance than a design that reduced the AP force on the post. This agrees with a retrieval study of several designs^1^ that reported that posts with a relatively wider ML dimension had increased damage on the medial and lateral surfaces.

The computational model demonstrated excellent agreement in predicting flexion angle of engagement and engagement velocity, while prior work demonstrated agreement in prediction of six-DOF TF and patellofemoral kinematics.^22^ Due to the resources and time requirements of experimental testing, it is impractical to perform large numbers of cadaveric simulations; we only tested one TKA device in four specimens. However, a limited number of tests allows for verification of the predictions from our computational simulations, which can subsequently be utilized for efficient, cost-effective comparison of multiple designs. When boundary conditions of the model were modified to better represent the physiological situation, there were changes in post-cam engagement angle and velocity. For instance, the same TKA component engaged ∼10° earlier due to the addition of relative hip-ankle motion. This highlights the importance of assessing joint loading mechanics under physiological boundary conditions that may not be feasible to evaluate experimentally. Computational simulations also facilitate comparison of contact mechanics, joint forces, and internal stresses that are usually infeasible to obtain from dynamic in vitro experiments. The model compares devices under the same soft-tissue constraint, alignment, and boundary conditions, allowing correlation between geometry in the absence of additional sources of variability.

We assessed engagement mechanics of these devices under a limited set of conditions. The analysis was performed for a single activity; alternative activities would test these components under different kinematic and loading conditions. However, a squat activity was chosen as an activity of daily living that required deep enough flexion to initiate post-cam engagement. Components were evaluated with a single set of representative soft-tissues. As illustrated by the standard deviation of the flexion angle at engagement for the four cadaveric specimens, substantial interspecimen variability exists in ligament integrity, and the level of constraint the soft-tissues provide to the knee influence kinematics and engagement mechanics. The anatomy and material properties used in the model were developed from another cadaveric specimen that was run in the KKS and exhibited kinematic behavior representative of a normal knee. Additional laxity tests were preformed on this specimen to calibrate ligament properties (attachment sites, initial tension, and stiffness) in the model.^22^ As such, the anatomy used in the model was representative of a typical specimen and was an appropriate platform for comparative analysis of components. However, to predict how these components would perform across a patient population, these devices should also be evaluated with a variety of soft-tissue representations to determine sensitivity to ligament laxity.

In conclusion, we verified the importance of condylar geometry, in addition to geometry of the post-cam mechanism itself, in controlling post-cam engagement mechanics during dynamic activity and compared post-cam engagement velocity for a variety of commercial TKA devices. This knowledge may guide implant design to incorporate condylar and post-cam geometric features that reduce edge loading and facilitate smooth post-cam engagement.
